# Effects of sleeve gastrectomy on rivaroxaban pharmacokinetics, efficacy, and safety

**DOI:** 10.3389/fphar.2026.1723656

**Published:** 2026-02-19

**Authors:** Saeed Alqahtani, Anfal Almutairi, Faten Aldajani, Manar Basudan, Arwa Alzahrani, Maha Alenazi, Emad Alsarhani, Abdulaziz Alsubaie, Alhassan Almaghrabi, Fahad Bamehriz, Hamad Alsubaie, Farjah Algahtani

**Affiliations:** 1 Department of Clinical Pharmacy, College of Pharmacy, King Saud University, Riyadh, Saudi Arabia; 2 Clinical Pharmacokinetics and Pharmacodynamics Unit, King Saud University Medical City, Riyadh, Saudi Arabia; 3 Department of Surgery, College of Medicine, King Saud University, Riyadh, Saudi Arabia; 4 Department of Medicine, College of Medicine, King Saud University, Riyadh, Saudi Arabia

**Keywords:** anticoagulants, bariatric surgery, pharmcokinetics, rivaroxaban, thromboprophylaxis

## Abstract

**Background:**

Thromboembolic events are potentially serious complications in patients undergoing bariatric surgery. Several studies have investigated the use of rivaroxaban in patients undergoing bariatric surgery. Further evidence is required to determine whether postsurgical anatomical and physiological changes affect the pharmacokinetics of rivaroxaban. This study aimed to investigate the pharmacokinetic, efficacy, and safety of rivaroxaban in patients who underwent sleeve gastrectomy surgery.

**Methods:**

Included patients who admitted for sleeve gastrectomy surgery and were scheduled to receive prophylactic doses of rivaroxaban. Pre- and post-operative rivaroxaban plasma concentrations were determined. Efficacy and safety were assessed 6 and 9 months after surgery. Results: Twenty patients (40% males) were included in the study. The average body weight was 117.6 ± 21.2 kg. The average AUC values before, after, and 7-day after bariatric surgery were 5.83 (1.23), 5.34 (1.87), and 6.29 (2.12) μg·h mL^−1^, respectively. The average C_max_ before, after, and 7-day after bariatric surgery were 0.45 (0.2), 0.37 (0.17), and 0.48 (0.23) μg mL^−1^, respectively. No significant differences were observed in the rivaroxaban PK parameters. No thrombosis events were reported over 6 or 9 months. In addition, 100% of the participants experienced no significant bleeding events or other adverse effects associated with rivaroxaban during the trial period.

**Conclusion:**

Rivaroxaban 10 mg shows promise as a potential medication for VTE prophylaxis after bariatric surgery. However, future studies with larger, more diverse populations are needed to confirm these findings and strengthen their applicability in clinical practice and to determine the optimal dosage and long-term safety profile in this patient population.

## Introduction

The health implications of obesity are myriad and multifaceted. Morbid obesity is rapidly becoming an increasing medical and socioeconomic burden. Consequently, the number of patients undergoing bariatric surgery is steadily increasing, and many of them require treatment for comorbidities, such as cardiovascular disease. The use of bariatric surgery to help patients lose weight is becoming more common (Rocha et al., 2006). Bariatric surgery leads to the most sustained reduction in weight and associated comorbidities; however, patients treated with bariatric surgery have an increased risk of venous thromboembolic events (VTE) (Rocha et al., 2006). Obesity is an independent risk factor for the development of venous thromboembolism, and the association between obesity and VTE after bariatric surgery is well established (Bartlett et al., 2015). Therefore, thromboembolic disease is a frequent complication in patients with morbid obesity, and long-term anticoagulation is often required (Almarshad et al., 2020; Thereaux et al., 2018). Venous thromboembolism is a major cause of postoperative morbidity and mortality in bariatric surgery (Almarshad et al., 2020; Thereaux et al., 2018). The reported postoperative incidence of VTE after bariatric surgery varies widely from 0.3% to 2.4% (Carvalho et al., 2023; Hamad and Choban, 2005; Haskins et al., 2015; Winegar et al., 2011).

Similarly, the rate of bleeding after surgery varies from 0% to 6% (Almarshad et al., 2020; Thereaux et al., 2018). This variation is likely due to differences in patient characteristics, type of bariatric surgery, dose of thromboprophylaxis (weight-adjusted vs. fixed-dose), duration of thromboprophylaxis, and measured outcomes (symptomatic vs. asymptomatic VTE) (Almarshad et al., 2020; Thereaux et al., 2018). VTE is a preventable disease, and thromboprophylaxis is a key strategy for reducing VTE-related mortality and morbidity after bariatric surgery (Almarshad et al., 2020; Thereaux et al., 2018).

Direct oral anticoagulants (DOACs) are used as alternatives to warfarin for the treatment of venous thromboembolism and the prevention of stroke or systemic embolism in patients with non-valvular atrial fibrillation (Julia and James, 2017; Vimalesvaran et al., 2018). The advantages of DOACs over warfarin include faster onset of action, fewer drug interactions, shorter half-life, wide therapeutic range, and lack of need for routine laboratory monitoring (Julia and James, 2017; Vimalesvaran et al., 2018). Since 2010, four DOACs (apixaban, dabigatran, edoxaban, and rivaroxaban) have been approved by the US Food and Drug Administration (FDA) for stroke prevention in nonvalvular atrial fibrillation and the acute treatment of VTE (Julia and James, 2017; Vimalesvaran et al., 2018).

Rivaroxaban is a direct factor Xa inhibitor recently approved in several countries for VTE treatment and stroke prevention in patients with atrial fibrillation (Vimalesvaran et al., 2018). It is rapidly absorbed in the upper GI tract, with a high bioavailability of 80%–100% (Vimalesvaran et al., 2018). Rivaroxaban has been shown to be effective and safe for the acute and extended treatment of VTE (Vimalesvaran et al., 2018). Theoretically, bariatric surgery has the potential to affect the pharmacokinetics of administered drugs via a multitude of mechanisms, and anatomical changes from bariatric procedures have several effects on drug absorption, which can have serious consequences for these patients (Moore and Kroll, 2017).

One possible consequence of bariatric surgery is a reduction in the bioavailability of oral medications, which occurs through multiple mechanisms, including altered absorption, distribution, metabolism, and/or elimination of orally administered drugs via changes in the gastrointestinal tract anatomy, body weight, and adipose tissue composition (Moore and Kroll, 2017).

Currently, data on the use of rivaroxaban, particularly in bariatric surgery, are limited. Nonetheless, some studies have indicated that it may be useful for preventing VTE following bariatric surgery. This study aimed to investigate the pharmacokinetic parameters of rivaroxaban as a prophylactic dose in obese patients undergoing sleeve gastrectomy surgery.

## Patients and methods

### Study design

A prospective single-center approach was used in this pharmacokinetic study to assess the effect of sleeve gastrectomy surgery on rivaroxaban pharmacokinetics. This study was conducted at King Saud University Medical City (KSUMC), Riyadh, Saudi Arabia.

### Participants

Adult patients (age ≥18 years) undergoing elective bariatric surgery were recruited for the study and consented. The inclusion criteria were the administration of rivaroxaban for thromboprophylactic anticoagulation and a body mass index (BMI) of at least 30 kg/m^2^. Patients with known rivaroxaban hypersensitivity, hepatic impairment (Child-Pugh class B or C), or significant renal impairment (creatinine clearance <30 mL/min) were excluded.

### Study procedure and rivaroxaban administration

Written informed consent was obtained from eligible subjects prior to study participation. Laboratory values, concurrent medication use, medical history, and baseline demographic information were recorded. As per their prescribed regimen, the participants were administered a single oral dose of rivaroxaban (10 mg once daily for 14 days). The post-operative rivaroxaban dose was administered on post-operative day 1, approximately 24 h after surgery, once patients were clinically stable and able to tolerate oral intake. At the time of post-operative dosing, patients were receiving a clear liquid diet in accordance with institutional post–sleeve gastrectomy protocols. Pharmacokinetic sampling was performed at three time points: pre-operatively, on post-operative day 1, and 7 days after surgery. On each occasion, blood samples were collected pre-dose and at 1, 2, 4, 6, 12, and 24 h following rivaroxaban administration. Venous blood samples were collected, and plasma was separated by centrifugation and stored at −80 °C until analysis.

### Study endpoints

The primary endpoint of this study was the PK parameters of rivaroxaban after oral administration—before, after, and 7 days post-surgery. The secondary endpoints included the assessment of efficacy and safety of rivaroxaban in patients at 6- and 9-month post-surgery. The efficacy outcome was a composite of deep vein thrombosis (DVT)—including both proximal and distal, as well as asymptomatic and symptomatic cases—or objectively confirmed pulmonary embolism (PE). The primary safety outcomes were major and non-major bleeding, as defined by the International Society on Thrombosis and Hemostasis (ISTH), including bleeding that led to transfusion or a decrease in hemoglobin levels of 2 g/dL during the intervention and observation periods (Schulman et al., 2010). The secondary safety outcomes were serious adverse effects of rivaroxaban, drug allergies, and drug sensitivity.

### Analytical assay

Plasma rivaroxaban concentrations were quantified using validated high-performance liquid chromatography as previously described (Alqahtani et al., 2023). A standard curve for rivaroxaban was prepared from healthy volunteers and spiked with rivaroxaban in the range of 10–10,000 ng/mL. The extraction yield was 97%. The method was valid, with a lower limit of quantification of 25 ng/mL. The intraday and interday coefficients of variation ranged from 0.6% to 5% when the spiked plasma samples were analyzed. Any data below the limit of quantification were to be handled using a mixed methods approach. However, all samples were above the limit of quantification.

### Statistics

To quantify the effect of sleeve gastrectomy on rivaroxaban exposure, AUC and C_max_ were natural log-transformed and analyzed using a bioequivalence-style approach. Within-subject comparisons were performed using paired contrasts on the log scale. Geometric mean ratios (GMR) and their 90% confidence intervals (CI) were obtained by back-transforming the mean differences in log-transformed parameters for Postop vs. Preop and 7-day Postop vs. Preop comparisons. Demographic and clinical features are summarized using descriptive statistics and presented in tabular form. When applicable, continuous variables are presented as the total number of measurements, mean, and standard deviation (interquartile range). Categorical variables are presented as frequencies and percentages.

### Ethical considerations and data management

The study protocol was approved by the Institutional Review Board (IRB) (Ref no: E-19-3748) of KSUMC in accordance with the principles outlined in the Declaration of Helsinki and Good Clinical Practice guidelines. Electronic data were collected and managed using a secure database. All research staff members received training in data collection techniques to guarantee precision and completeness.

## Results

### Participant characteristics

Twenty patients who underwent elective laparoscopic sleeve gastrectomy were included in this study. Table 1 summarizes the demographic and clinical characteristics of the study population. The mean age of the participants was 33.9 ± 10 years, of which 40% were male. The mean body weight was 117.6 ± 21.2 kg and BMI was 41.1 ± 5.9 kg/m^2^.

**TABLE 1:** Baseline demographic and disease characteristics of patients included in the analysis (n = 20).

Characteristics	Mean (SD)
Age, years	33.9 (10)
Sex, % male/%female	40/60
Weight, kg	117.6 (21.2)
Height, cm	167.8 (8.7)
BMI, kg/m^2^	41.1 (5.9)
Serum creatinine, mmol/L	65.8 (12.2)
Albumin concentration	37.8 (2.8)
AST	18.9 (9.6)
ALT	36.1 (16.8)
Total bilirubin	8.7 (3)
INR	0.94 (0.05)
PT	12.97 (0.8)
aPTT	34.28 (3.4)

### Rivaroxaban pharmacokinetics

#### Pre- and post-bariatric surgery comparison

**FIGURE 1:**
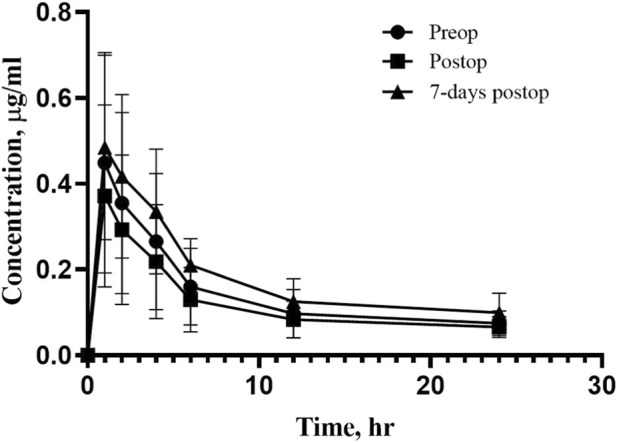
Individual rivaroxaban concentrations (µg/mL). The bar represents the average concentration for each group.

Figure 1 shows the rivaroxaban concentrations before and after bariatric surgery. The rivaroxaban area under the curve (AUC) before bariatric surgery was 5.83 μg·h mL^−1^ compared to 5.34 μg·h mL^−1^ post-operative day 1 and 6.29 μg·h mL^−1^ 7-day after the surgery. There was a slight decrease in C_max_ after surgery 0.37 μg mL^−1^ compared to 0.45 μg mL^−1^ before the surgery. The C_max_ recovered to 0.48 μg mL^−1^ 7-day after the surgery. PK parameters are summarized in Table 2. When analyzed using a bioequivalence-style approach, rivaroxaban exposure was largely preserved after sleeve gastrectomy. The GMR for AUC was 0.94 (90% CI: 0.80–1.11) for Postop vs. Preop and 1.12 (90% CI: 0.91–1.38) for 7-day Postop vs. Preop. For C_max_, the GMR was 0.83 (90% CI: 0.82–0.84) post-operative day 1 and 1.20 (90% CI: 1.09–1.32) at 7 days Postop compared with Preop values, indicating a transient reduction in peak concentration immediately after surgery with recovery by day 7 (Table 3). Apparent elimination half-life values were comparable across study periods; however, these estimates should be interpreted cautiously given the limited characterization of the terminal phase. Median T_max_ values were similar across the three study periods; however, given the discrete sampling schedule, T_max_ represents an observed value within the sampling intervals rather than a precisely estimated parameter.

**TABLE 2:** Pharmacokinetic parameters for patients; preop, postop day 1, and 7-day postop the mean (SD) is presented.

Parameters	Preop	Postop	7-day postop
AUC (μg·h mL^−1^)	5.83 (1.23)	5.34 (1.87)	6.29 (2.12)
C_max_ (μg mL^−1^)	0.45 (0.20)	0.37 (0.17)	0.48 (0.23)
t_1/2_ (h)	23 (3.5)	26 (4.2)	20 (4.7)
V_ss_/f (L)	48.3 (5.7)	65.7 (7.4)	40.2 (4.8)
T_max_ (h)	1 (0.3)	1 (0.4)	1 (0.2)

t_1/2_ values represent apparent elimination half-life estimates derived from the available sampling window.

**TABLE 3:** The GMR and CI for AUC and C_max_; preop, postop day 1, and 7-day postop.

Parameter	Comparison	GMR	90% CI
AUC	Postop day 1 vs. Preop	0.94	0.80–1.11
	7-day Postop vs. Preop	1.12	0.91–1.38
C_max_	Postop day 1 vs. Preop	0.83	0.82–0.84
	7-day Postop vs. Preop	1.20	1.09–1.32

### Safety and efficacy

All patients were followed up for 6 and 9 months after bariatric surgery. No significant major or non-major bleeding events were observed during the trial period. In addition, no serious adverse effects or drug allergies associated with rivaroxaban were reported. No thromboembolic or major bleeding events were observed during the 6- and 9-month follow-up period; however, the study was not powered to detect differences in clinical event rates.

## Discussion

Low-molecular-weight heparin (LMWH) is the treatment of choice for thromboprophylaxis in patients who have undergone bariatric surgery because of its ease of administration and consistent pharmacokinetics. However, growing evidence supports the potential effectiveness of rivaroxaban as a thromboprophylaxis after bariatric surgery. Compared to LMWH, rivaroxaban has several advantages, such as oral delivery, no requirement for frequent monitoring, and improved patient adherence. This enhances patient comfort and simplifies postoperative care.

Studies have shown that rivaroxaban is safe and effective in preventing blood clots in patients after bariatric surgery (Kroll et al., 2018; Kroll et al., 2023; Kroll et al., 2017). The 2021 ISTH SSC communication conditionally recommend rivaroxaban as VTE prophylaxis after bariatric surgery in high-risk patients (Martin et al., 2021). However, they suggested starting with a parenteral anticoagulant in the immediate postsurgical phase, followed by a transition to rivaroxaban or another DOAC in the stable post-acute phase (Martin et al., 2021). It is important to note that these are general guidelines and that individual patient factors should always be considered when determining the most appropriate VTE prophylaxis strategy (Martin et al., 2021).

Drug absorption is a concern after bariatric surgery, particularly in surgeries involving substantial anatomical changes in the gastrointestinal tract (Moore and Kroll, 2017). Variations in medication absorption after bariatric surgery have been observed in previous studies and may affect the effectiveness of rivaroxaban. Therefore, the current study investigated the pharmacokinetics of rivaroxaban in patients undergoing bariatric surgery to elucidate the impact of surgical interventions on drug absorption and distribution. In addition, these patients were followed up at 6 and 9 months to determine the efficacy and safety of rivaroxaban.

Our findings showed that although peak rivaroxaban concentrations are transiently reduced immediately following sleeve gastrectomy, overall exposure remains largely unchanged, with normalization of peak levels within the first postoperative week. Although no thrombotic or major bleeding events were observed during follow-up period, this finding should be interpreted with caution. The present study was not powered to detect clinically differences in these outcomes, given the relatively low incidence of venous thromboembolism and major bleeding. Therefore, the absence of events should be considered exploratory and hypothesis-generating rather than confirmatory of clinical safety or efficacy. Several factors may explain these findings. Rivaroxaban has high permeability and is primarily absorbed in the upper small intestine. Additionally, previous studies have reported that rivaroxaban has nearly 100% bioavailability at a 10 mg dose, minimizing the impact of bariatric surgery on the absorption process (Mueck et al., 2014).

Notably, the post-operative pharmacokinetic evaluation was carried out on the post-operative day 1, which is marked by physiological alterations as decreased oral intake and changed gastrointestinal motility. Therefore, the observed pharmacokinetic parameters at this time point should be interpreted within this clinical context. Furthermore, the stated T_max_ represents the observed sampling time of maximum concentration rather than a precisely estimated peak due to the fact that blood sampling was carried out at predetermined discrete time periods. This is an intrinsic limitation of the study design.

The early post-operative phase is marked by limited oral intake, decreased gastric emptying, and possible gastrointestinal symptoms such nausea or vomiting, even though rivaroxaban 10 mg is less dependent on food for absorption than higher doses. These variables might affect how tablets dissolve and disintegrate, as well as be a contributor in the higher interindividual variability seen in pharmacokinetic parameters, especially C_max_, on post-operative day 1. The recovery of peak concentrations by day 7 suggests that these effects are transient and diminish as oral intake and gastrointestinal function normalize.

An important observation in the present study was the increase in inter-individual variability in rivaroxaban exposure during the early post-operative period, as reflected by the higher variability in AUC values compared with the pre-operative phase. Heterogeneity in postoperative gastrointestinal physiology, such as variations in gastric emptying, intestinal motility, oral intake tolerance, and recovery of absorptive capacity after sleeve gastrectomy, may be the cause of this increased variability. From a clinical perspective, increased inter-individual variability in exposure may translate into a higher risk of under- or over-exposure in certain patients, particularly in the immediate post-operative setting. Notably, variability appeared to decrease by post-operative day 7, indicating that these effects are temporary and go away as oral intake and gastrointestinal function stabilize.

These findings are consistent with those of previous studies. Kroll et al. recently published a randomized clinical trial on the safety and efficacy of rivaroxaban as thromboprophylaxis in patients after bariatric surgery (Kroll et al., 2023). The patients were administered 10 mg of oral rivaroxaban once daily for 7 or 28 days (Kroll et al., 2023). A composite of DVT and PE was the main efficacy outcome, and major bleeding, clinically relevant non-major bleeding, and mortality were the main safety outcomes (Kroll et al., 2023). In general, the use of 10 mg rivaroxaban was effective and safe in the early postoperative phase after bariatric surgery in both the short and long prophylaxis groups (Kroll et al., 2023).

Kroll et al. published two phase I clinical studies (Kroll et al., 2018; Kroll et al., 2017). In their first study, they investigated the effects of two types of bariatric surgery–gastric bypass and sleeve gastrectomy–on the pharmacokinetic and pharmacodynamic parameters of rivaroxaban (Kroll et al., 2017). They found no significant differences in the pharmacokinetic and pharmacodynamic parameters of rivaroxaban 1 and 3 days after either type of surgery (Kroll et al., 2017). In the second study, they extended their investigations on the effect of bariatric surgery on the pharmacokinetic and pharmacodynamic parameters of rivaroxaban 6 and 8 months after surgery (Kroll et al., 2018). There were no significant differences between the two surgical procedure groups (Kroll et al., 2018).

In a recent study, Leven et al. evaluated the pharmacokinetics and safety of full-dose rivaroxaban (20 mg) in post-bariatric surgery patients (Leven et al., 2024). Compared to controls, patients who underwent Roux-en-Y gastric bypass or sleeve gastrectomy exhibited reduced rivaroxaban absorption, resulting in lower drug exposure (Leven et al., 2024). Despite these variations, the differences were minor and unlikely to be clinically significant given the interindividual variability seen in the general population (Leven et al., 2024).

In 2013, Mahlmann et al. reported in a case report the pharmacokinetics of rivaroxaban in a patient with a high venous thromboembolism risk and unstable INR after recent bariatric surgery (Mahlmann et al., 2013). The course of plasma concentrations of rivaroxaban 20 mg are as follows: after 3 h, a peak concentration of 224.22 ng/mL was observed with a slow decrease until a trough level of 35.54 ng/mL after 24 h (Mahlmann et al., 2013), and after the next dose of rivaroxaban, a peak level of 262.46 ng/mL was measured (Mahlmann et al., 2013). The patients’ INR immediately increased (INR after 3 h was 3.86) and remained elevated throughout the day, with a slow decrease until trough levels were reached (6, 12, and 24 h values of 2.93, 2.90, and 2.42, respectively). After the next rivaroxaban dose, an immediate increase in the INR was observed (Mahlmann et al., 2013). These data indicate that the standard dose of rivaroxaban (20 mg od) is immediately absorbed and affects the standard coagulation parameters in this population (Mahlmann et al., 2013).

Real-world studies have also investigated the efficacy and safety of DOAC after bariatric surgeries (Kushnir et al., 2019; Langworthy et al., 2023). Kushnir et al. reviewed 102 patients’ charts prescribed apixaban or rivaroxaban after bariatric surgery and found no evidence of recurrent VTE in the apixaban group and 1 (1.7%) in the rivaroxaban group (Kushnir et al., 2019). Langworthy et al. investigated the efficacy and safety of DOAC (apixaban, dabigatran, and rivaroxaban) in 191 patients with a history of bariatric surgery (Langworthy et al., 2023). Clotting and bleeding events occurred in 11 (5.8%) and 42 (22%) patients, respectively, with a statistically significant increased risk of bleeding in the rivaroxaban vs. apixaban group (Langworthy et al., 2023).

The advantage of this study is that it reports both pharmacokinetic changes in rivaroxaban along with its safety and efficacy in bariatric surgery, unlike previous reports that mainly focused on one aspect. The current study provides further evidence of the efficacy and safety of use 10 mg of rivaroxaban in patients after bariatric surgery. However, the main limitations of this study is its relatively small sample size, which may affect the generalizability of the findings to a broader patient population. A limited sample size can reduce statistical power, increasing the risk of variability and potential bias. This may lead to challenges in detecting true differences or associations, particularly for secondary endpoints. Additionally, the small number of participants may not fully capture variations in patient characteristics, comorbidities, or treatment responses, which could influence the outcomes. Future studies with larger, more diverse populations are needed to confirm these findings and strengthen their applicability in clinical practice. It is important to note that we monitored pharmacokinetic parameters on days 1 and 7 after surgery to ensure that there were no changes over time. In addition, we monitored the efficacy and safety outcomes for up to 9 months after surgery.

## Conclusion

Rivaroxaban 10 mg shows promise as a potential medication for VTE prophylaxis after bariatric surgery. This study provides evidence supporting the addition of rivaroxaban as a convenient thromboprophylactic option for patients. The decision to use rivaroxaban should be made on an individual basis, considering the patient’s specific risk factors, potential benefits, and drawbacks. However, Future studies with larger, more diverse populations are needed to confirm these findings and strengthen their applicability in clinical practice and to determine the optimal dosage and long-term safety profile in this patient population.

## Data Availability

The raw data supporting the conclusions of this article will be made available by the authors, without undue reservation.
